# Unraveling the genetic mysteries of spinal muscular atrophy in Chinese families

**DOI:** 10.1186/s13023-024-03523-0

**Published:** 2025-01-15

**Authors:** Shanshan Gao, Duo Chen, Qianqian Li, Xuechao Zhao, Chen Chen, Lina Liu, Miao Jiang, Zhenhua Zhao, Yanhua Wang, Xiangdong Kong

**Affiliations:** 1 The Genetics and Prenatal Diagnosis Center, The Department of Obstetrics and Gynecology, The First Affiliated Hospital of Zhengzhou University, Jianshe Rd, Erqi District, Zhengzhou, 450052 Henan China; 2 NHC Key Laboratory of Birth Defects Prevention, Institute of Reproductive Health, Henan Academy of innovations in medical Science, The northeast corner of the Intersection of Huanghai Road and Biotechnology 2nd Street, Airport District, Zhengzhou, 451163 Henan China

**Keywords:** Spinal muscular atrophy, 5q SMA, Non-5q SMA, *SMN1* gene, Genetic diagnosis, Prenatal diagnosis

## Abstract

**Objective:**

Spinal muscular atrophy (SMA) is a motor neuron disorder encompassing 5q and non-5q forms, causing muscle weakness and atrophy due to spinal cord cell degeneration. Understanding its genetic basis is crucial for genetic counseling and personalized treatment options.

**Methods:**

This study retrospectively analyzed families of patients suspected of SMA at our institution from February 2006 to March 2024. Various molecular techniques, including multiplex ligation-dependent probe amplification analysis, long-range polymerase chain reaction (PCR) combined with nested PCR, Sanger sequencing, and whole-exome sequencing were employed to establish a thorough genetic variant profile in 680 Chinese pedigrees with clinically suspected SMA.

**Results:**

Out of 680 families suspected of having SMA, 675 exhibited mutations in the *SMN1* gene, while three families were linked to mutations in the *IGHMBP2* gene. One family exhibited a genetic variation in the *NEB* gene, and another family exhibited a variation in the *SCO2* gene. Among the families with mutations in the *SMN1* gene, 645 families exhibited either E7‒E8 or E7 homozygous deletion. Some families displayed E7‒8 heterozygous deletions along with other mutations, such as E1 or E1‒6 heterozygote deletion and point mutations. Furthermore, one family demonstrated a compound-heterozygous double mutation, while another carried a type “2 + 0” mutation alongside a point mutation.

**Conclusions:**

This study comprehensively analyzed the genetics of suspected familial SMA cases in the Chinese population, providing insights into the molecular genetic mechanisms of SMA and the utility of various detection techniques. The findings revealed important implications for genetic counseling, prenatal diagnosis, and targeted therapies in clinical practice.

**Supplementary Information:**

The online version contains supplementary material available at 10.1186/s13023-024-03523-0.

## Introduction

Spinal muscular atrophy (SMA) is a hereditary disorder characterized by the degeneration of anterior horn cells, leading to muscle weakness and atrophy [1, 2]. Various types of SMA, such as 5q and non-5q SMA, have been linked to a range of genetic mutations.

The most severe clinical and genetic form of SMA, known as 5q SMA, is linked to mutations in the *SMN1* gene located on chromosome 5q13.2. This autosomal recessive genetic disorder encompasses various subtypes, including type 0, type 1 (Werdnig-Hoffmann disease), type 2 (Dubowitz syndrome), type 3 (Kugelberg-Welander disease), and type 4 (adult-onset SMA) [3–6]. The global prevalence of this disease is estimated to be 1 in 10,000 individuals, with a carrier frequency of 1 in 50 in the general population [3, 7]. 5q SMA was historically the most common genetic cause of infant mortality; however, this changed in 2016 with the advent of three novel treatments: Nusinersen, Zolgensma, and Risdiplam [3].

Non-5q SMA is characterized by its infrequency, genetic heterogeneity, clinical variability, and lower prevalence compared to 5q SMA [8]. This condition is linked to pathogenic gene mutations such as *IGHMBP2*, *PLEKHG5*, *GDAP1*, *ASAH1*, *ATP7A*, *TRPV4*, *VCP*, and others [2, 8–10]. Progress in diagnostic methodologies has resulted in an increasing number of clinically diagnosed cases. Currently, an established treatment is lacking for non-5q SMA.

The advancement of technology has led to an increasing array of genetic testing methods for detecting SMA. These methods differ in complexity, cost, reliability, and speed. Approximately 95% of individuals with 5q SMA exhibit a deletion of exon 7 of the *SMN1* gene or the c.840C > T variation between *SMN1* and *SMN2* [1, 11]. The literature discusses various detection methods, including fluorescent quantitative PCR, multiplex ligation-dependent probe amplification (MLPA), PCR-restriction fragment length polymorphism, PCR denaturing high-performance liquid chromatography and digital PCR (dPCR), among others [11–17]. The integration of long-range PCR and nested PCR, or third-generation sequencing, has been employed to identify the residual 5% of 5q SMA cases caused by point mutations [17, 18]. Furthermore, next-generation sequencing methods, including whole-exome sequencing (WES), have effectively detected non-5q SMA cases [8, 10].

The genetic background of SMA can be complex, and the absence of a large family cohort hampers a comprehensive understanding of condition [1]. In this research, we presented the largest cohort of SMA in China and shared the insights acquired through the diagnostic process. This result significantly contributes to the global SMA genetic dataset and enhances testing protocols. This study addressed what subsequent genetic analysis clinicians should perform upon suspecting SMA in a patient.

## Materials and methods

### Study cohort

We retrospectively studied 680 families, including patients suspected of SMA, at the Genetics and Prenatal Diagnostic Center, First Affiliated Hospital of Zhengzhou University, from February 2006 to March 2024. All patients underwent a comprehensive physical examination conducted by an experienced neurologist. The inclusion criteria are as follows: the presence of at least one clinical manifestation of myasthenia, muscular atrophy, joint deformity, or scoliosis; the absence of sensory and other neurological abnormalities; and normal or slightly elevated serum creatine phosphokinase levels. This study was approved by the Ethics Committee of the First Affiliated Hospital of Zhengzhou University (Ethics number: KS-2018-KY-36). All participants provided voluntary informed consent before their inclusion.

Pregnant women underwent villus sampling at 12‒14 weeks or amniotic fluid retrieval at 16‒20 weeks for a prenatal diagnosis. The decision to undergo prenatal diagnosis was entirely voluntary for any family.

### DNA extraction

DNA was extracted from human peripheral blood, amniotic fluid, and chorionic villi using the Qiagen Genomic DNA Extraction Kit or a tissue extraction protocol on an Eppendorf epMotion 5075 m workstation. The DNA concentration was determined using the Life Technologies Qubit dsDNA HS Assay Kit.

### MLPA

The number of *SMN1* and *SMN2* gene copies was determined using the SALSA P060-B2 SMA Kit (MRC-Holland, Amsterdam, Netherlands). DNA was denatured at 98 ℃ for 5 min. For probe hybridization, the sample was heated to 95℃ for 1 min, then incubated at 60 ℃ for 16–24 h. The hybridization probe was ligated at 54 ℃ for 15 min, followed by 98 ℃ for 5 min to inactivate the ligase. PCR amplification involved 30 cycles of 95 ℃ for 30 s, 60 ℃ for 30 s, and 72 ℃ for 30 s, with a final extension at 72 ℃ for 20 min. PCR products were analyzed on an ABI 3130 Genetic Analyzer and processed with Coffalyzer software. Ratios falling below 0.7, between 0.7 and 1.3, 1.3 and 1.7, and between 1.7 and 2.3 indicated one, two, three, and four gene copies, respectively.

### Long-range PCR, nested PCR, and sanger sequencing

Long-range PCR was performed using the KOD FX kit (KFX-101, TOYOBO, Japan) to amplify a 28.2-kb region containing exons 1‒8 of *SMN1* by step-down cycle PCR in a 25 µL reaction volume, with 12.5 µL of 2 × PCR Buffer, 0.4 mM of each dNTP, 0.15 µM of each primer (SMN_F and SMN_R), 0.5 U of polymerase and 50 ng of genomic DNA. Long-range PCR was performed as follows: initial denaturation at 94 ℃ for 2 min, followed by five cycles of denaturation at 98 ℃ for 10 s and annealing/extension at 71.2 ℃ for 15 min, followed by 5 cycles of.

denaturation at 98 ℃ for 10 s and annealing/extension at 69.2 ℃ for 28 min, followed by 5 cycles of denaturation at 98 ℃ for 10 s and annealing/extension at 67.2 ℃ for 28 min, and eight cycles of denaturation at 98 ℃ for 10 s and annealing/extension at 65.2 ℃ for 28 min, with a final extension at 65.2 ℃ for 15 min. Subsequently, nested PCR was used to amplify each *SMN1* exon using 1 µL of the initial product as a template. Amplification of each *SMN1* exon was performed with TIANGEN PCR kit in a 25 µL reaction volume, with 12.5 µL of 2 × Taq PCR Master Mix, 0.4 µM of each primer, and 1 µL of template. Nested PCR was performed under the following conditions: initial denaturation at 95 ℃ for 4 min, followed by 32 cycles of denaturation at 95 ℃ for 30 s, annealing and extension at 60 ℃ for 30 s, and extension at 72 ℃ for 30 s, with a final extension at 72℃ for 7 min. All primer sequences are displayed in Supplementary Table 1. The resulting product was purified and sequenced using the ABI 3130 Genetic Analyzer [18].

### WES

Qubit 4.0 (Thermo Fisher Scientific Inc., USA) was used to quantify genomic DNA, and DNA libraries were constructed with the Ada/Index Kit (UDI for LIM) and Enzyme Plus Library Prep Kit (iGeneTechCo., Ltd, Beijing, China). Exome coding and splicing regions were captured using the AIExome Human Exome Panel V2 Plus with TargetSeq One Hyb & WashKit (iGeneTech Co., Ltd, Beijing, China) following the manufacturer’s instructions. Subsequently, the captured libraries were sequenced on the NovaSeq6000 platform (Illumina, San Diego, CA, USA) with an average sequencing depth of over 100 × and a sequencing coverage of over 98% at 20 × . Genome Analysis Toolkit’s best practice guidelines for aligning paired-end reads were followed using Sentieon (release 20,808.05). Duplicate reads were removed with Picard version 2.9.0, according to GATK guidelines. Databases such as the 1000 Genomes Project, dbSNP, the Genome Aggregation Database, ClinVar, the Human Genomic Mutation Database and OMIM annotated the variants. To validate the genotypes of the proband, parents, and other family members, all candidate gene variants were confirmed by Sanger sequencing.

### dPCR

A BioDigital-QING dPCR™ system was used (Turtle Tech Ltd., Shanghai, China). The BioDigital-QING Loader™ generated one layer of water-in-chamber droplets inside the microfluidic chip. After droplet generation, the chips were amplified by BioDigital-QING Cycler™ using 50 ℃ for 10 min, 95 ℃ for 5 min, 50 cycles of 94 ℃ for 20 s, and 56 ℃ for 40 s, and finally held at 25 ℃. After PCR, the chips were placed in a BioDigital-QING Imager™ to detect fluorescence amplitude signals. The number and ratio of droplets containing different fluorescence signals were counted, followed by result calculation using Poisson distribution.

## Results

### An overview of the enrolled families

A total of 680 Chinese families, including patients suspected of SMA, were involved in the study. The genes involved and the types of mutations are presented in Table 1. A total of 675 families exhibited an association with the *SMN1* gene, while the *IGHMBP2* gene was linked to three families, and the *SCO2* and *NEB* genes were each identified in one family. The mutations identified in the *IGHMBP2* gene were E628* and A786Pfs*45, K220R and R637L, and R443C and A600V, respectively. The mutations identified in *SCO2* were F237fs and A97_Q101dup, while those identified in *NEB* were R8358Vfs*20 and E8167*.

**Table 1:** Summary of genetic variation data for 680 families

Gene	NM	Variation type	Number of families	Proportion (%)	
*SMN1*	NM_000344	Homozygote deletion in E7–8 or E7	645	94.8	99.3
		E7‒8 heterozygote deletion combined with E1 heterozygote deletion	1	0.1	
		E7‒8 heterozygote deletion combined with E1‒6 heterozygote deletion	1	0.1	
		Heterozygote deletion in E7‒8 accompanied by single-point mutation	26	3.8	
		“2 + 0” genotype accompanied by single point mutation	1	0.1	
		Compound-heterozygote mutations	1	0.1	
*IGHMBP2*	NM_002180	Compound-heterozygote mutations	3	0.4	0.7
*SCO2*	NM_005138	Compound-heterozygote mutations	1	0.1	
*NEB*	NM_001164508	Compound-heterozygote mutations	1	0.1	

E7–8 indicates that exons 7 and 8

### Families related to the *SMN1* gene

**Fig. 1:**
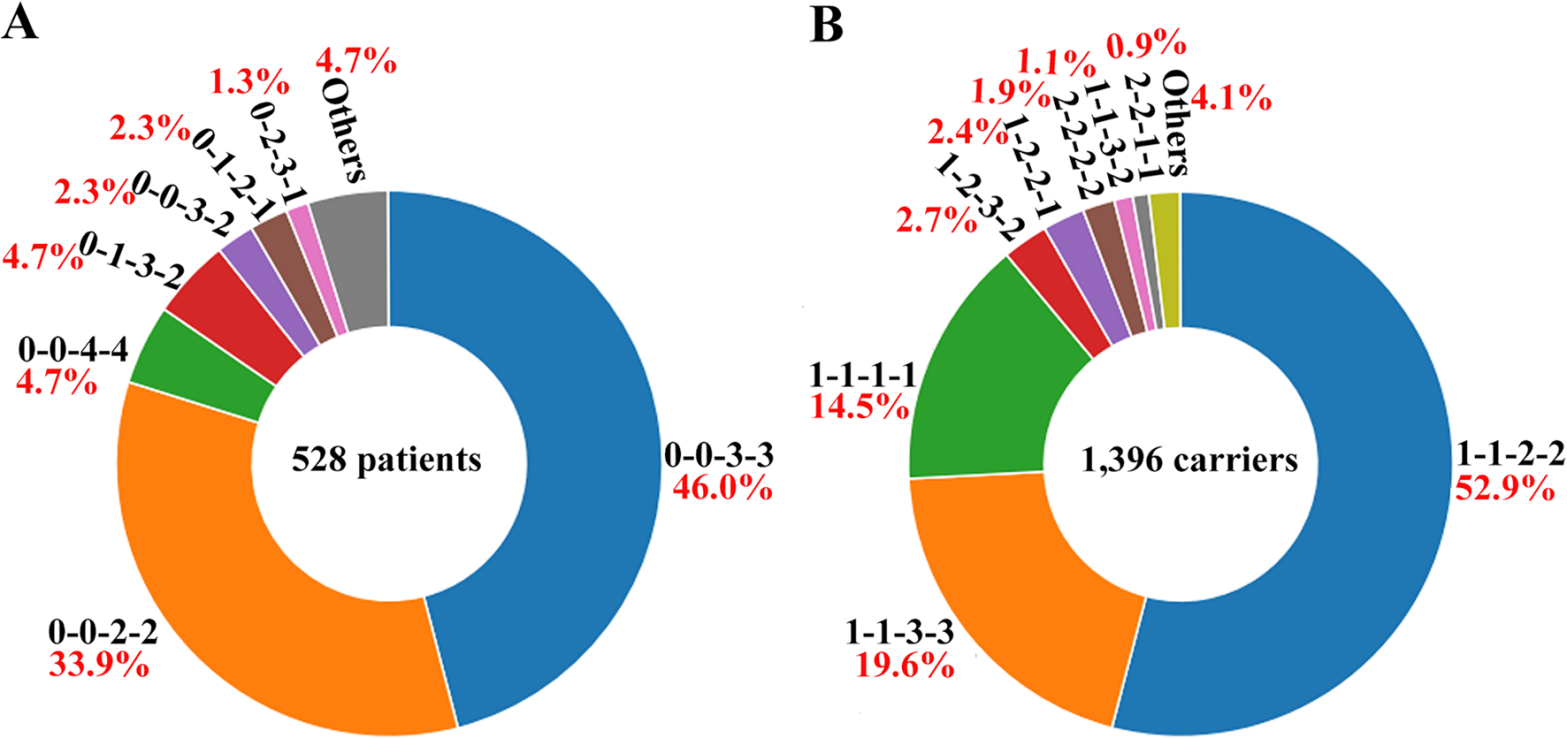
MLPA results for 645 Chinese families with homozygous deletions in *SMN1* E7‒8 or E7 regions. **A** Analysis of genotypes in 528 patients and affected fetuses. **B** Analysis of carrier genotypes in 1396 parents and sibling fetuses or children. The “0-0-3-3” genotype indicates that the copy number of exon 7 of the *SMN1* gene is 0, the copy number of exon 8 of the *SMN1* gene is 0, the copy number of exon 7 of the *SMN2* gene is 3, and the copy number of exon 8 of the *SMN2* gene is 3, respectively

Our research revealed that a significant proportion of families (94.8%, 645/680) exhibited homozygous deletion in *SMN1* E7‒8 or E7, as depicted in Table 1 and Fig. 1. Only two families exhibited E7‒8 heterozygous deletion combined with E1 or E1‒6 heterozygote deletion. Among the remaining families (4.1%, 28/680), point mutations were observed in *SMN1*. Notably, one family displayed a compound-heterozygous double mutation (Table 1).

In a cohort comprising 645 Chinese families with a homozygous deletion in *SMN1* E7‒8 or E7, the predominant patient genotypes were identified as 0-0-3-3 (46.0%), 0-0-2-2 (33.9%), and 0-0-4-4 (4.7%), as illustrated in Fig. 1A. The most common carrier genotypes, as depicted in Fig. 1B, were 1-1-2-2 (52.9%), 1-1-3-3 (19.6%), and 1-1-1-1 (14.5%). The genotypes of 2-2-2-2 and 2-2-1-1 may indicate carriers of the “2 + 0” allele.

**Fig. 2:**
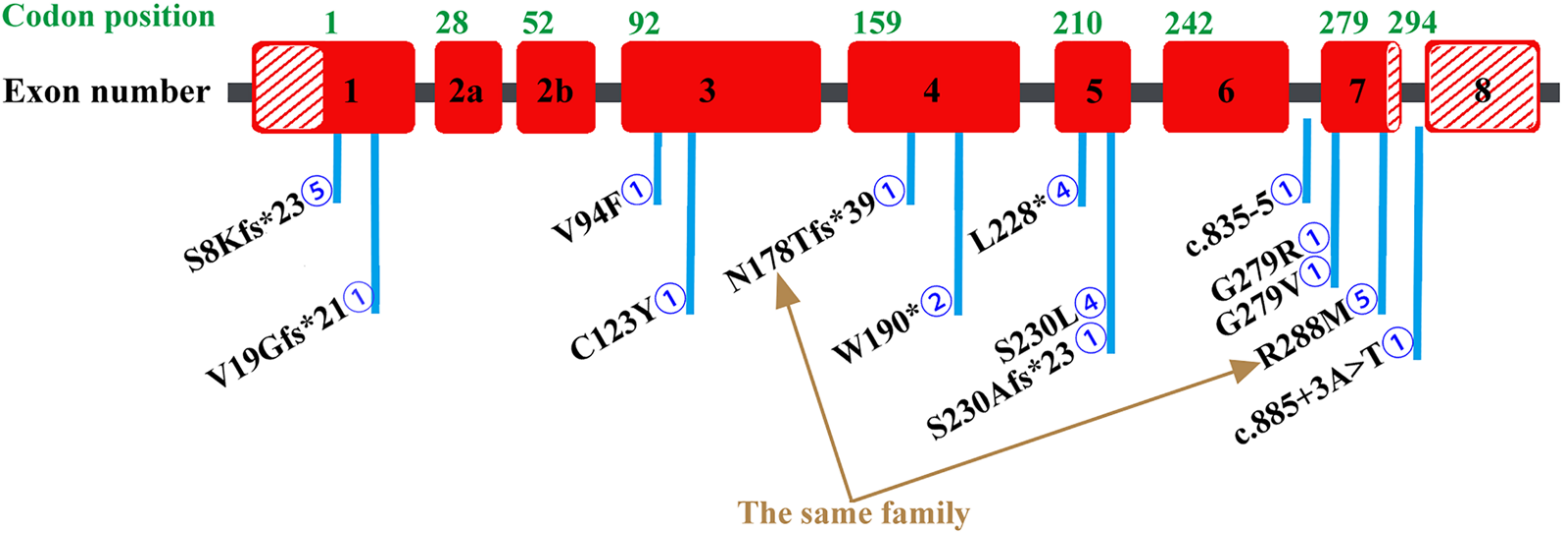
Point mutation data for the *SMN1* gene (the blue numbers indicate the number of mutant families)

Among the 28 families with point mutations in *SMN1*, 14 distinct mutations were identified, as illustrated in Fig. 2. The inheritance of point mutations from parents was identified in affected patients and fetuses without observing any de novo mutations. About 26 families exhibited heterozygous deletion in exon 7, along with a single-point mutation (Fig. S1). One family demonstrated a compound-heterozygous double mutation comprising the variants N178Tfs*39 and R288M (Fig. S2). Another family was identified as carriers of type “2 + 0” and the S8Kfs*23 mutation (Fig. S3). The mother possesses a genotype of 2-2-1-1 with an S8Kfs*23-point mutation, while the father possesses a “2 + 0” genotype of 2-2-1-1. The patient exhibits a genotype of 1-1-2-2 with an S8Kfs*23-point mutation, and the brother has a genotype of 3-3-1-1 with the same S8Kfs*23-point mutation.

### Asymptomatic persons with homozygous deletion of *SMN1* E7

**Fig. 3:**
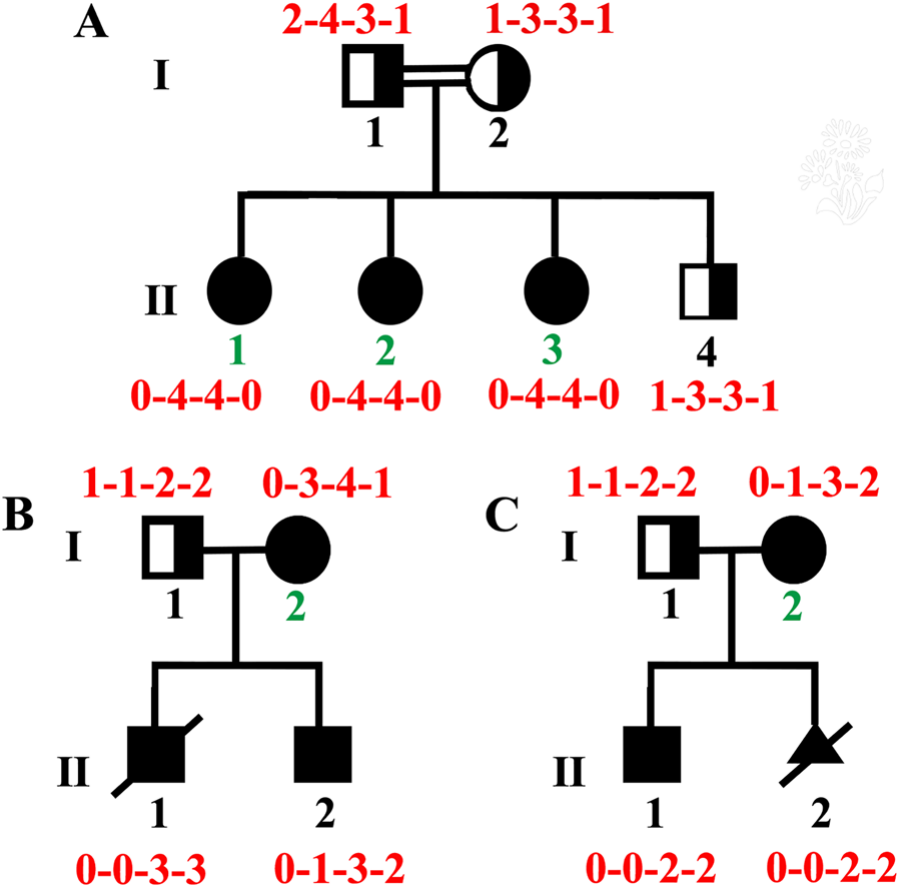
Family pedigrees affected by SMA depict *SMN1* E7 homozygous deletion in unaffected subjects. The MLPA results are shown in the figure

Five asymptomatic individuals were identified in three separate families, denoted by green numbers (Fig. 3), all of whom exhibited homozygous deletion of exon 7 of the *SMN1* gene. In Family A, a 40-year-old pregnant woman (II 2) underwent genetic disease carrier screening due to consanguinity between her parents. The screening revealed a homozygous deletion of the *SMN1* gene E7. Subsequent MLPA analysis of the family members indicated that the older sister (42 years old; II 1) and the younger sister (32 years old; II 3) shared the same genotype as the pregnant woman (II 2). The mother (I 2) and brother (II 4) were carriers of SMA, while the father (I 1) was likely a carrier of SMA “2 + 0” (Fig. 3A). In Family B, the father (I 1) was identified as a carrier, while the mother (I 2), who was 50 years old in 2024, displayed no symptoms. The firstborn son (II 1) succumbed to SMA at the age of five, while the younger son (II 2), who is presently 16 years old, is confined to a wheelchair (Fig. 3B). In Family C, the father (I 1) was identified as a carrier, while the mother (I 2), aged 30 in 2024, exhibited no symptoms. The eldest son (II 1), aged six years, who cannot ambulate, received two injections of Nusinersen. The second fetus was terminated due to SMA (Fig. 3C).

### Fascinating discoveries during the detection

**Fig. 4:**
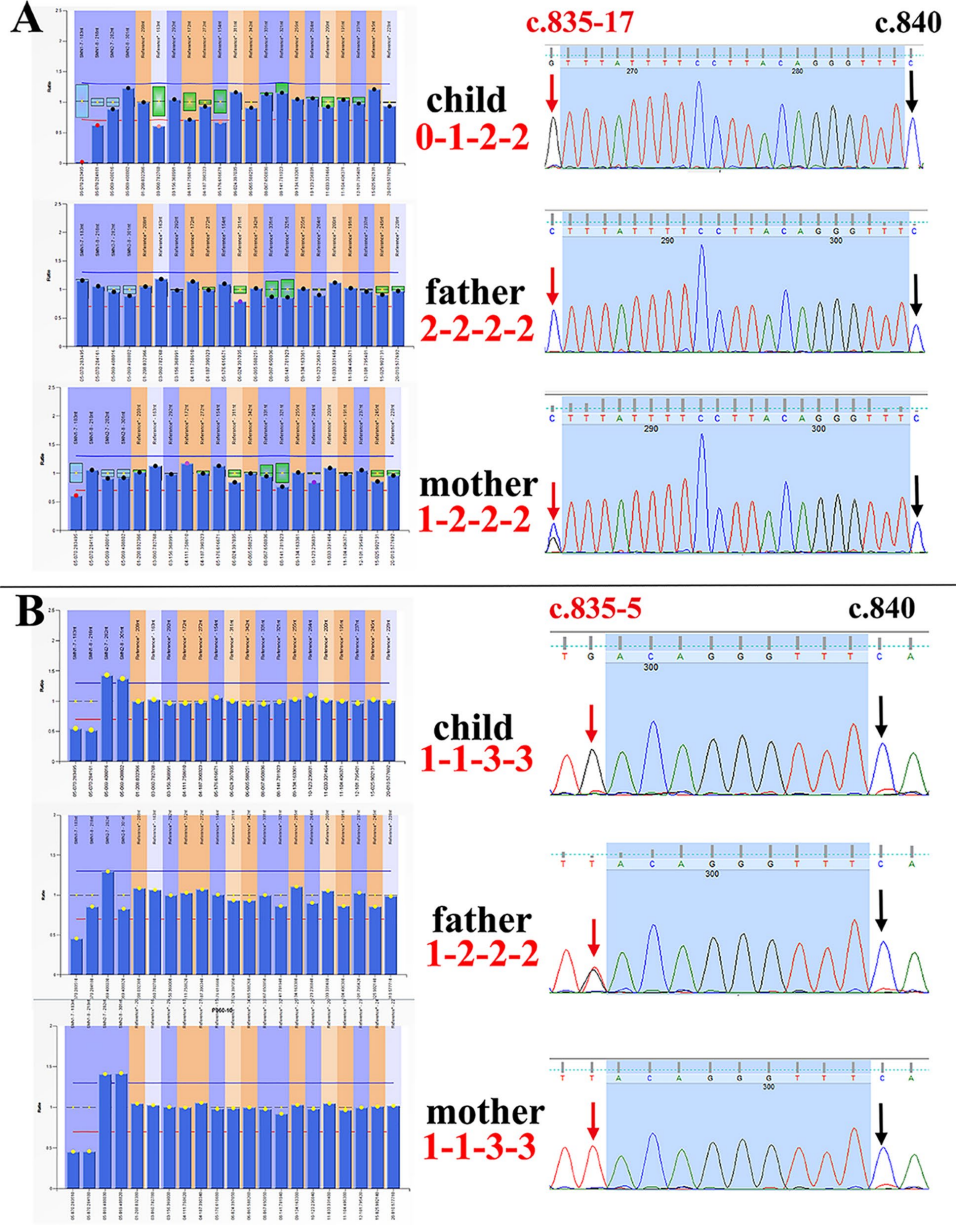
MLPA analysis produced false-positive results in two families

Two families were identified as having false positives for MLPA (Fig. 4). In Family A, the child exhibited dysphagia, a choking cough, and muscle weakness since birth. The MLPA analysis confirmed the diagnosis of SMA in the child, attributing it to a homozygous deletion of the *SMN1* gene in E7. The mother was identified as a “1 + 0” carrier, while the father was presumed to be a carrier of the “2 + 0” type. However, dPCR and the sequencing results superseded the MLPA results. dPCR results revealed that the child exhibited heterozygous deletion of the *SMN1* gene in E7. Meanwhile, the sequencing results revealed that the nucleotide at position c.840 was cytosine, which indicates the presence of the *SMN1* gene in the child (Fig. 4A). A single copy of the *SMN1* gene was detected in the child, featuring a benign variant c.835-17C > G, leading to a diagnosis of carrier status for SMA. The mother was identified as a “1 + 1” gene type. Subsequent WES results revealed R8358Vfs*20 and E8167* mutations in the *NEB* gene in the child. Both variants are inherited from the maternal and paternal lineages, respectively. The fetus in Family A also carried the same two disease-causing mutation sites of the *NEB* gene. In Family B, the father's MLPA results were inconsistent with the results of sequencing and dPCR. The MLPA analysis revealed the presence of a single copy of *SMN1* E7, while the sequencing and dPCR revealed the presence of two copies (Fig. 4B). The c.835-5 T > G of the *SMN1* gene is pathogenic.

**Fig. 5:**
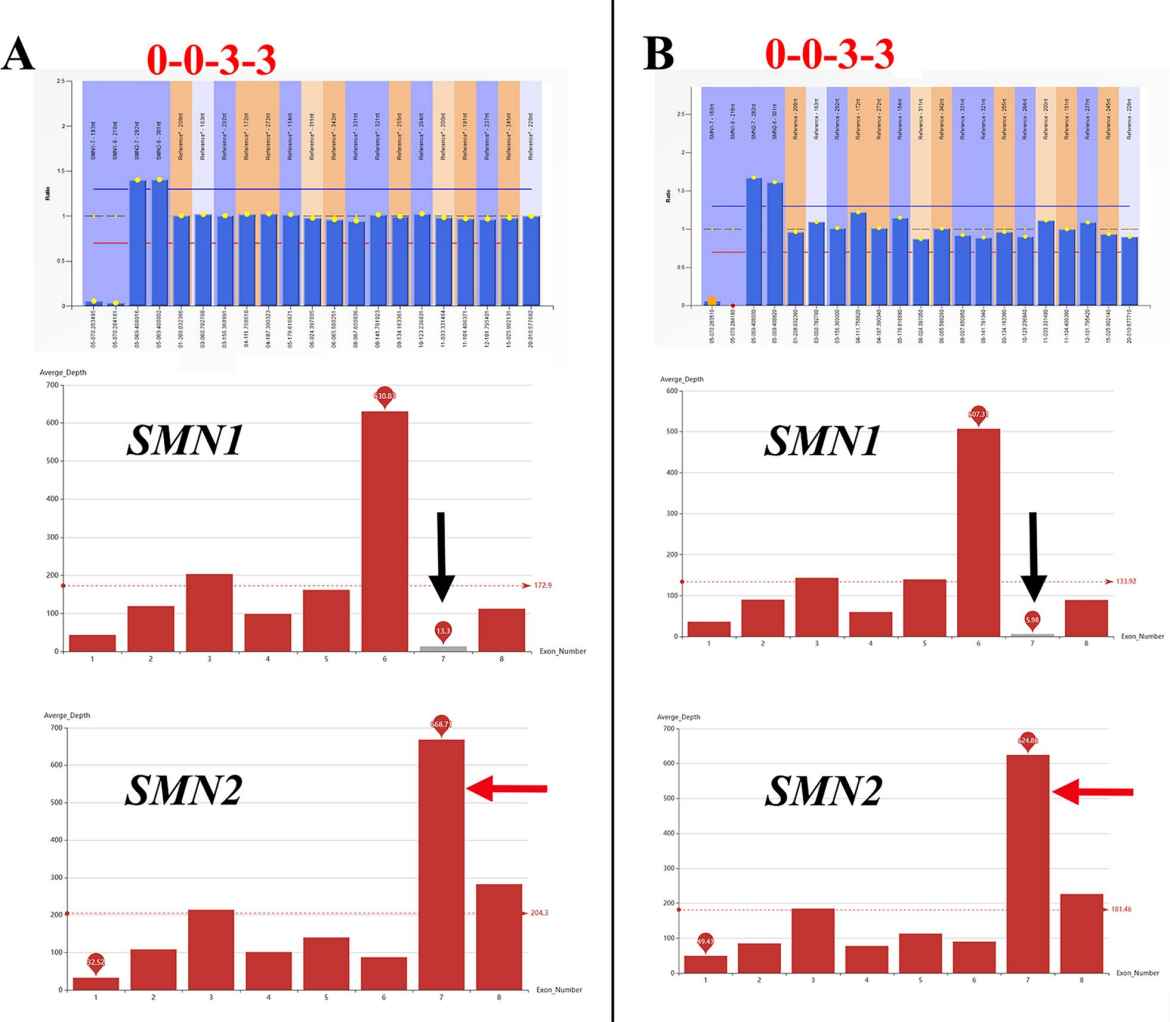
MLPA and WES analyses were performed on two SMA-afflicted children

Two children diagnosed with SMA underwent both MLPA and WES in two separate families (Fig. 5). The MLPA results displayed a pattern of 0-0-3-3 for both individuals. The sequencing depth of the *SMN1* gene exon 7 in WES analysis was observed to be below the mean, denoted by a black arrow, while the *SMN2* gene exon 7 exhibited a higher sequencing depth, as denoted by a red arrow.

## Discussion

This study provided a comprehensive analysis of the genetic profile of SMA and presented the most extensive dataset currently known to us. It also discusses the challenges faced during testing and proposes follow-up testing protocols for individuals suspected of SMA and their family members.

In addition to the presence of the *SMN* gene in most families, other genes were identified. Mutations within the immunoglobulin mu DNA binding protein (*IGHMBP2*), an RNA–DNA helicase, lead to SMA with respiratory distress type I (SMARD1) and Charcot-Marie-Tooth type 2S (CMT2S) [19]. The *IGHMBP2* gene causes non-5q SMA. Furthermore, mutations in the *SCO2* gene result in cytochrome c oxidase deficiency, causing symptoms such as Leigh syndrome, hypertrophic cardiomyopathy, lactic acidosis, ventilator insufficiency, and a phenotype similar to SMA [20]. A few dozen cases of the *SCO2* gene have been reported worldwide. This study expanded the mutation spectrum of *SCO2* genes. Linear myopathy associated with the *NEB* gene is a rare congenital neuromuscular disorder characterized by muscle weakness, hypotonia, delayed acquisition of gross motor skills, and diminished respiratory capacity [21]. In this study, the family harboring the *NEB* gene mutation was incidentally associated with a false positive result in the MLPA assay for the *SMN1* gene.

The genotypic diversity observed among patients with 5q-SMA indicates the presence of structural variability, including partial gene deletions, point mutations, duplications, or conversions within the *SMN1* and *SMN2* genes. This report represents a preliminary investigation into Exon 1‒6 deletions (Table 1), identified using the MLPA P021 probe. The genotype 0-0-3-3 exhibits the highest prevalence among patients, while the genotype 1-1-2-2 is most frequently observed among carriers (Fig. 1). It is widely acknowledged that carriers of the genotype 1-1-2-2 give birth to individuals with the genotype 0-0-2-2, as opposed to 0-0-3-3. According to the findings, the majority (73.83%) of the 149 Chinese patients with *SMN1* deletion possessed two copies of *SMN2* [22]. However, the prevalence of the 0-0-2-2 genotype was not the highest in this study. This may be attributed to the death of the SMA-afflicted children (0–0–2–2 genotype) in most families during prenatal diagnosis visits, which were not included in the statistics, resulting in the underrepresentation of this genotype.

Genotypes such as 0–1-3-2, 0–2-3-1, 1–2-3-2, 1-2-1-0, and others may indicate the presence of single-nucleotide polymorphisms of *SMN2* and *SMN2*/*SMN1* hybrid genes [23]. In families with homozygous deletion of exon 7, most parents are carriers with only one copy of *SMN1*, while a minority exhibits the presence of two copies of *SMN1* (Fig. 1). Our previous research has demonstrated that some fathers possessing two copies exhibit a normal “1 + 1” genotype, while others display a “2 + 0” genotype [12]. Kirwin et al. reported a homozygous double mutation in an SMA patient whose parents were consanguineous, specifically identified as p.Val19fs*24 and p.Pro221Leu [24]. In this research, we documented the presence of a heterozygous double mutation of N178Tfs*39 and R228M in an SMA patient whose parents were not blood-related (Fig. 2 and S2). Moreover, the N178Tfs*39 variant is inherited from the father, while the R228M variant is from the mother.

The variability in the copy number and structural composition of the *SMN2* gene among individuals may contribute to the observed heterogeneity in the clinical presentation and severity of patients with 5q-SMA [25]. Previous studies suggest that the number of *SMN2* copies is inversely related to the severity of the disease, and the presence of the *SMN1*/*SMN2* hybrid gene is associated with patients exhibiting a relatively mild disease course [26, 27]. Discrepancies in the number of E7 and E8 segments within the *SMN1* or *SMN2* gene, as detected by MLPA analysis, may indicate the formation of hybrid genes. Sanger sequencing analysis identified a mutation in the *SMN2* gene (NM_01741.3) at position c.*239A > G in the pregnant individual (II 2) from Family A, as depicted in Fig. 3 (Fig. S4). Typically, locus c.*239 of Exon 8 is G in *SMN1* and A in *SMN2*. Therefore, the presence of hybrid genes and high copy numbers may explain the asymptomatic status observed in some individuals (Fig. 3). Although the boy (II 2) from Family B and the mother (I 2) from Family C exhibit similar MLPA results, their disease manifestations differ. These findings suggest that additional factors, such as mutations, splicing modifiers, and epigenetic modifications, that may influence the severity of SMA [28–30]. Females with asymptomatic *SMN1* deficiency exhibit elevated expression levels of plastin 3 (PLS3), a phenomenon that has been demonstrated to ameliorate axonal growth abnormalities in animal models of *SMN1* deficiency [31]. Consequently, it is imperative for future research endeavors to devise novel methodologies and approaches to comprehensively elucidate the genetic variations that underlie the diverse clinical manifestations of SMA.

The MLPA technique is widely utilized globally for detecting E7-deficient SMA. In this investigation, two false positives were detected during the MLPA analysis (Fig. 4). The occurrence of a false positive result may be attributed to the binding region of the MLPA-P060 upstream probe within the nucleotide positions c.835-30 to c.840 of *SMN1* E7 (a total of 35 nucleotides) (https://www.mrcholland.com). If a mutation occurs within this specific region of the DNA of the individual under examination, excluding the c.840 site, the detection probe will fail to bind to the target DNA, resulting in a loss of signal. Therefore, it is crucial to meticulously analyze the MLPA-P060 results by considering various scenarios, including the presence of minor ratios or signals, the absence of a concurrent decrease in the 7/8 exon signal of *SMN 1/2*, and inconsistencies in the 7/8 exon sum of *SMN 1/2*.

Child A, aged seven months, exhibited a lack of exertion in the lower extremities (Fig. 5). Initially, one pediatrician recommended WES for the child; however, after two weeks, a negative result was ultimately achieved. Two months after the initial examination, another pediatrician suggested the possibility of SMA in Child A. We immediately conducted MLPA and sequencing assays targeting the c.840 site to confirm the diagnosis of SMA after two days. Concurrently, the authors reviewed all WES data for Child A and identified changes in the sequencing depth of *SMN1/2* E7. Child B, aged 14, was under suspicion for Duchenne muscular dystrophy (DMD) by the neurologist; however, subsequent testing using MLPA-P034/035 and WES produced negative results. Given the changes in the sequencing depth of *SMN1/2* E7 in WES data, we suspected that he might have SMA, which was later confirmed by MLPA-P060.

SMA diagnosis is initially established through clinical manifestations and confirmed by genetic analysis (Fig. S5). A definitive genetic diagnosis is crucial for genetic counseling, prenatal diagnosis, and targeted therapeutic interventions. For timely intervention for SMA families, it is imperative to promptly establish a diagnosis. Given the notable prevalence of deletions of *SMN1* E7, it is recommended to prioritize the detection of deletions, particularly at the c.840 site. It is speculated that if the *SMN1* gene is greater than or equal to 2 copies, 5q-SMA can be excluded. However, our research indicates the presence of two copies of *SMN1* in patients, and the possibility of 5q-SMA cannot be definitively ruled out (Fig. S5). Each detection method has its own limitations. Therefore, it is essential to integrate clinical symptoms with a comprehensive analysis of multiple detection methods. Furthermore, SMA diagnosis requires interdisciplinary cooperation among healthcare professionals and laboratory personnel from different departments, such as pediatrics, prenatal diagnostic centers, obstetrics, and neurology.

This study also has several limitations. First, the cohort was drawn from a single institution, potentially introducing selection bias and limiting the generalizability of the findings to the broader Chinese population or other populations globally. Second, the study does not address how the identified mutations correlate with clinical phenotypes or disease severity. It will be intriguing to observe the impact of these point mutations on the functionality and stability of the SMN protein. This will be the focal point of our future research.

In conclusion, SMA poses a significant challenge for clinicians and scientists due to its extensive variability in clinical symptoms and genetic features. This study offers a comprehensive genetic analysis of suspected familial SMA cases in the Chinese population. The results enhanced our understanding of the molecular genetic mechanisms underlying SMA and the utility of different molecular detection techniques, providing significant implications for genetic counseling, prenatal diagnosis, and targeted therapeutic strategies in clinical practice.

## Supplementary Information

Additional file 1: Figure S1. Family pedigrees of 26 families exhibiting an E7-8 heterozygous deletion accompanied by a single-point mutation in the SMN1 gene. The letter M stands for mutation.

Additional file 2: Figure S2. Genotype of the family with a compound-heterozygous double mutation. The letter M denotes mutation.

Additional file 3: Figure S3. The genotype of each family member was identified as a carrier of type "2+0" or the S8Kfs*23 mutation. The letter M stands for mutation.

Additional file 4: Figure S4. The Sanger sequencing analysis of the pregnant individual from Figure 3A.

Additional file 5: Figure S5. Flowchart for SMA genetic testing and counseling.

Additional file 6.

## Data Availability

All data generated or analysed during this study are included in this published article [and its supplementary information files].

## Abbreviations

SMA: Spinal muscular atrophy
MLPA: Multiplex ligation-dependent probe amplification
PCR: Polymerase chain reaction
WES: Whole-exome sequencing
IGHMBP2: Immunoglobulin mu DNA binding protein
SMARD1: SMA with respiratory distress type I
CMT2S: Charcot-Marie-Tooth type 2S
PLS3: Plastin 3
DMD: Duchenne muscular dystrophy
