# Supplementary figures and images for: Unraveling the genetic mysteries of spinal muscular atrophy in Chinese families
Source: Orphanet J Rare Dis. 2025 Jan 15;20:25. doi: 10.1186/s13023-024-03523-0 (PMC11734480; doi:10.1186/s13023-024-03523-0)

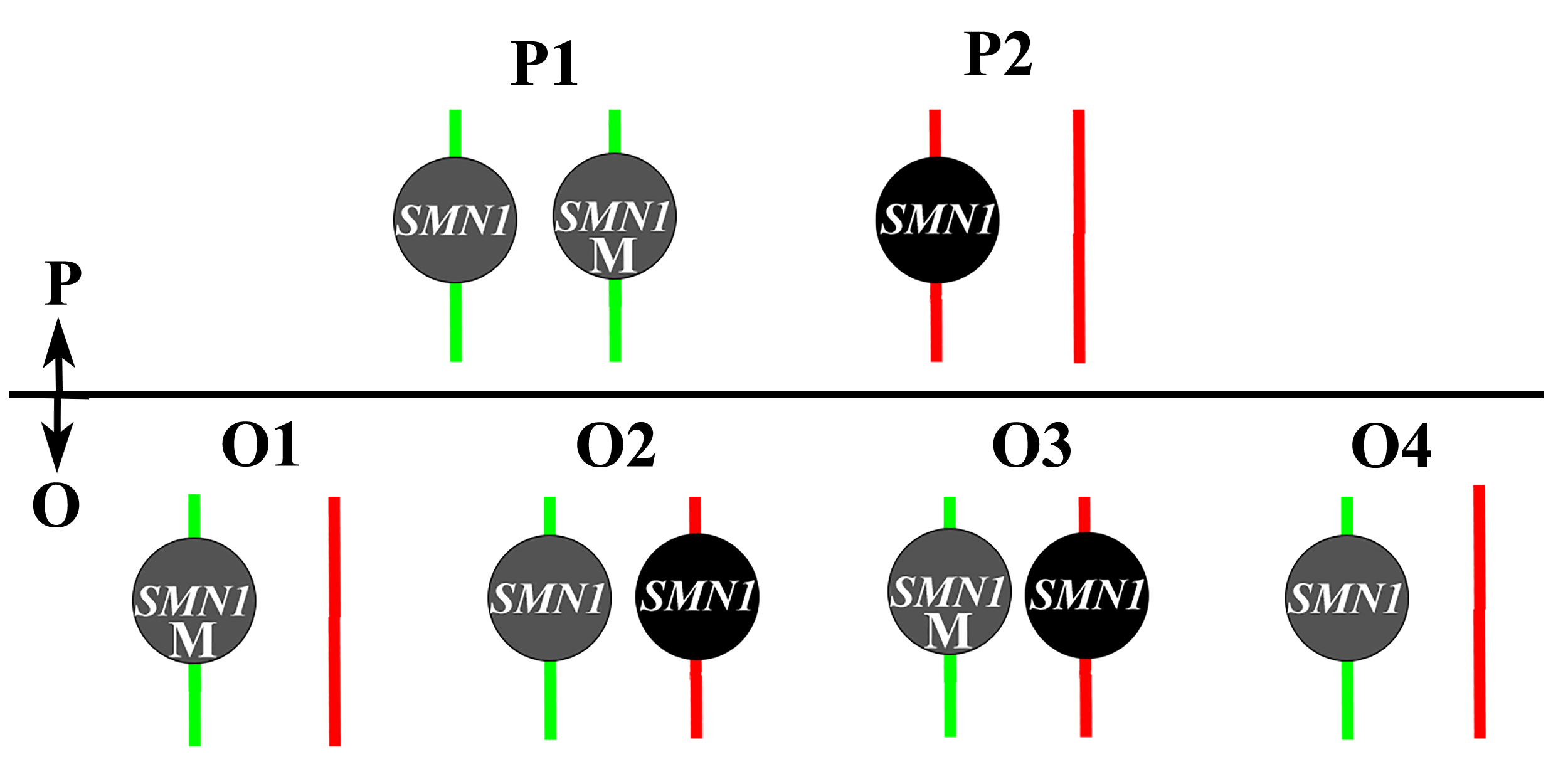

Supplement: Supplementary file 1 — Additional file 1: Figure S1. Family pedigrees of 26 families exhibiting an E7-8 heterozygous deletion accompanied by a single-point mutation in the SMN1 gene. The letter M stands for mutation. [file 13023_2024_3523_MOESM1_ESM.tif]

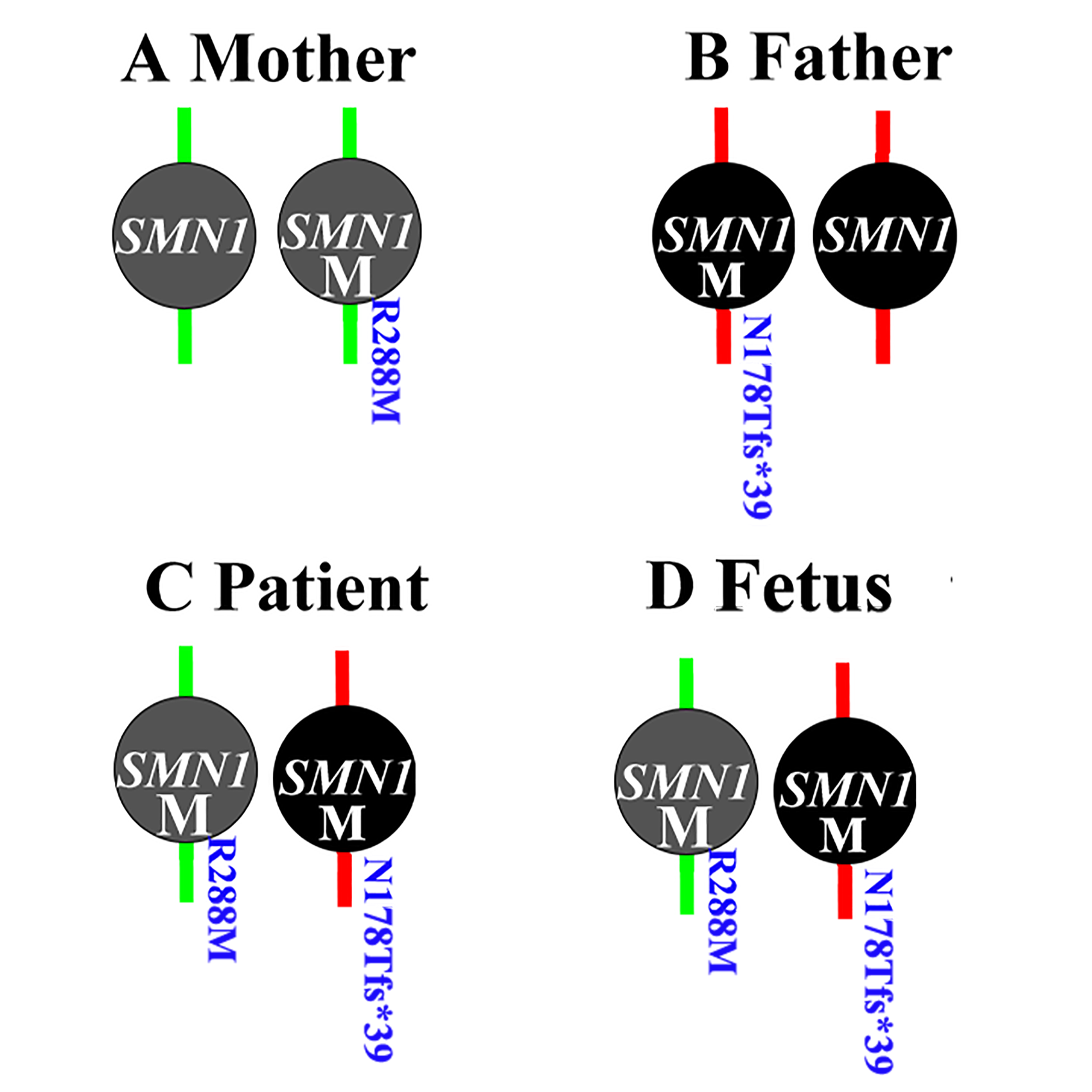

Supplement: Supplementary file 2 — Additional file 2: Figure S2. Genotype of the family with a compound-heterozygous double mutation. The letter M denotes mutation. [file 13023_2024_3523_MOESM2_ESM.tif]

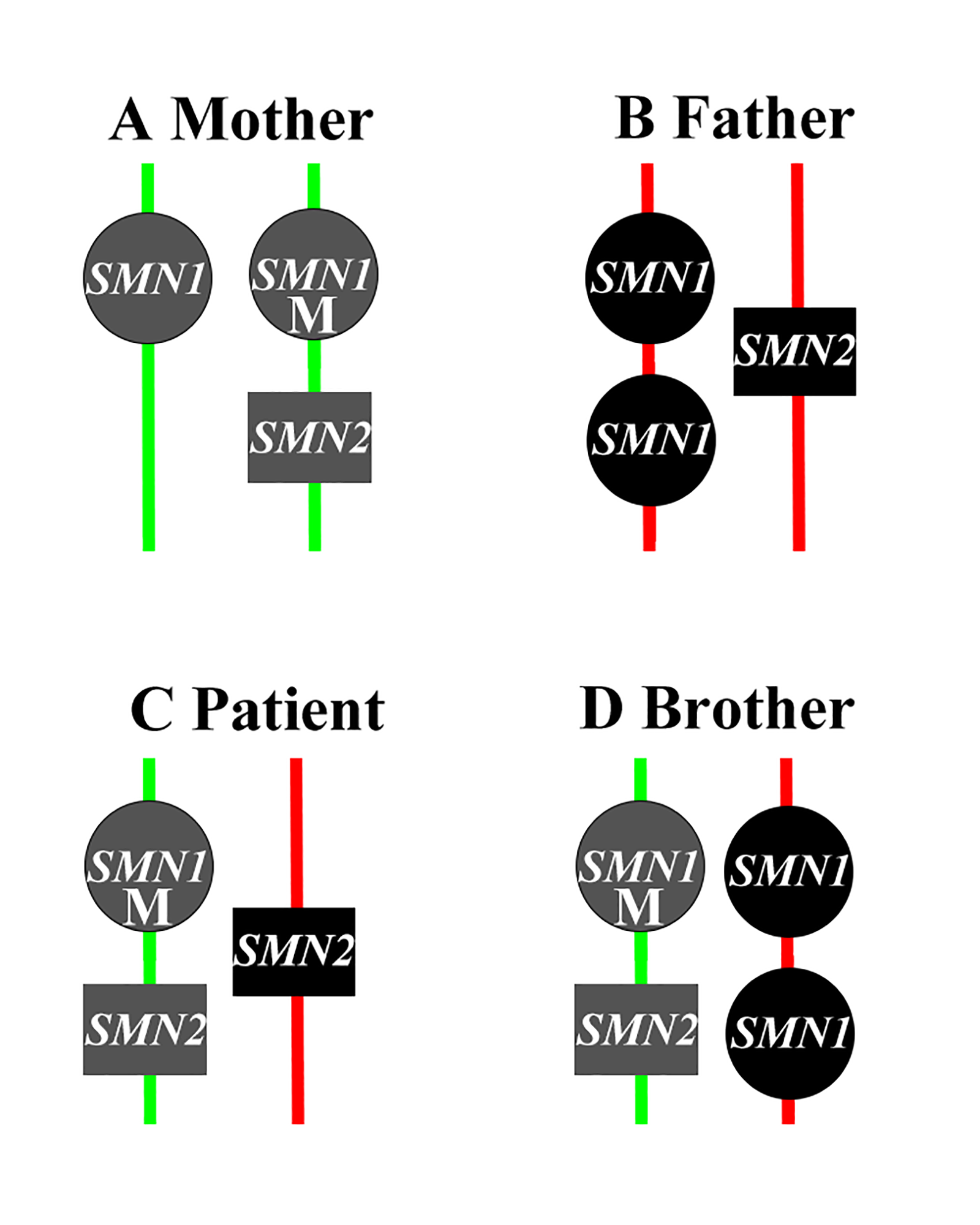

Supplement: Supplementary file 3 — Additional file 3: Figure S3. The genotype of each family member was identified as a carrier of type "2+0" or the S8Kfs*23 mutation. The letter M stands for mutation. [file 13023_2024_3523_MOESM3_ESM.tif]

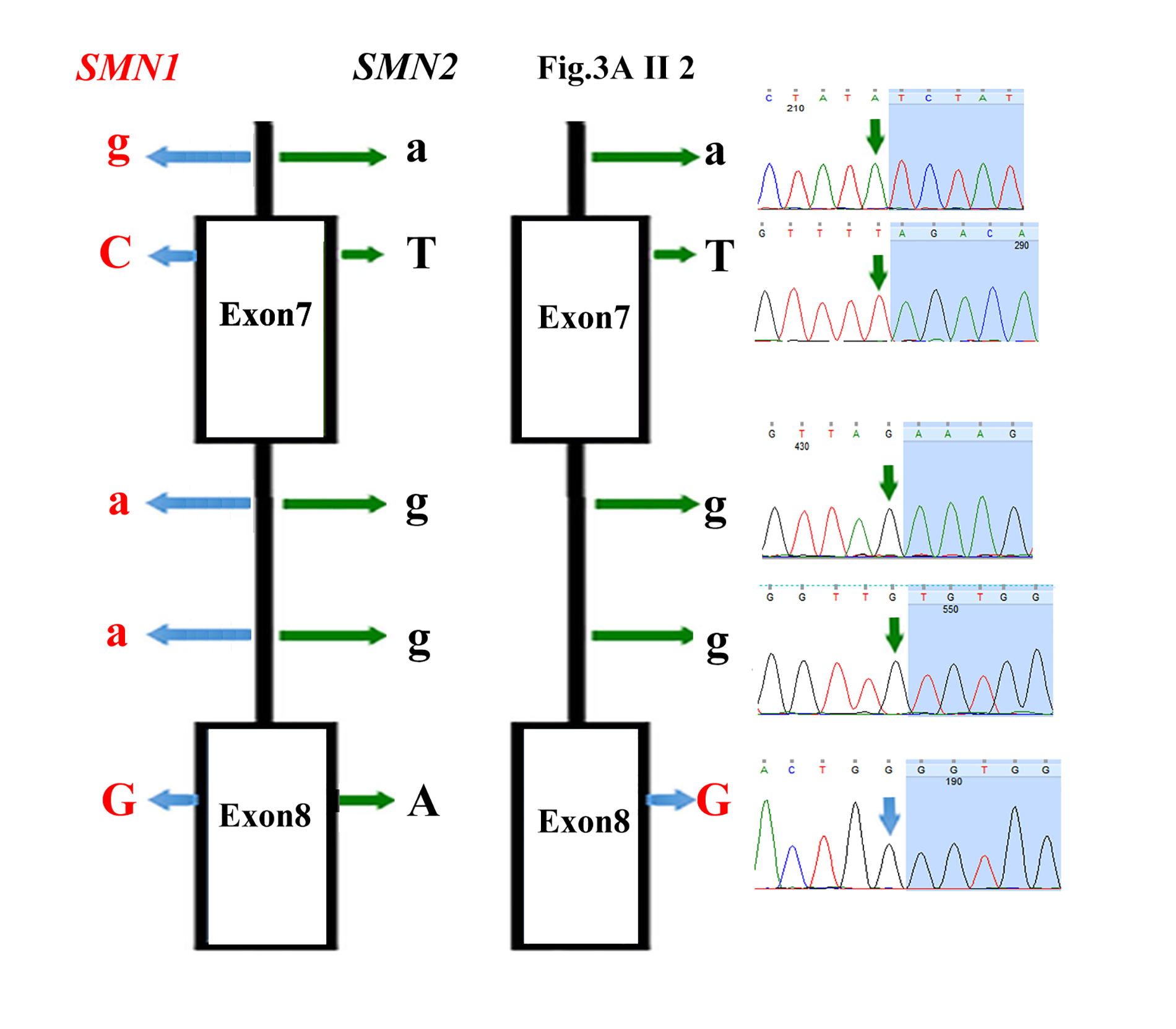

Supplement: Supplementary file 4 — Additional file 4: Figure S4. The Sanger sequencing analysis of the pregnant individual from Figure 3A. [file 13023_2024_3523_MOESM4_ESM.tif]

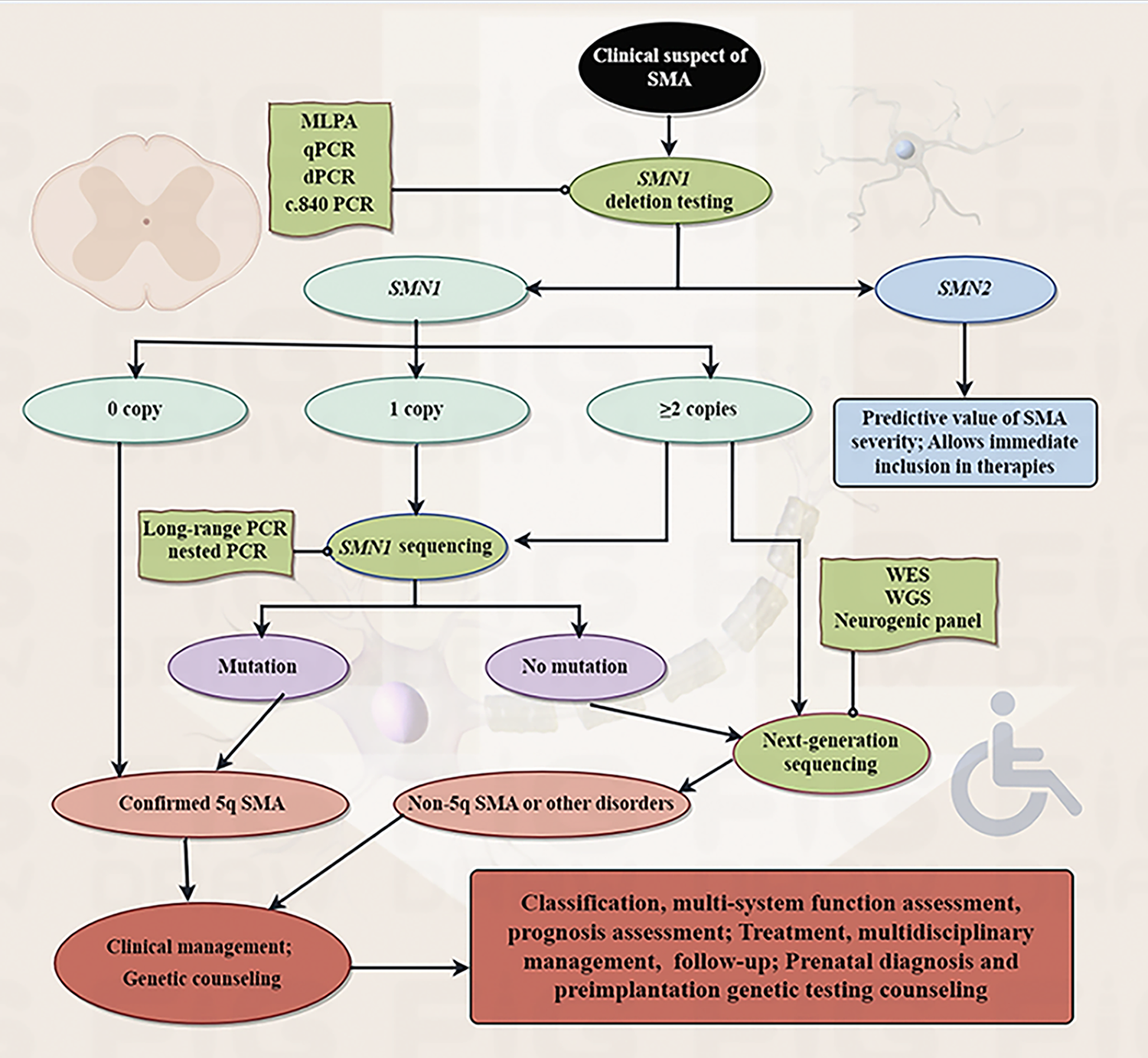

Supplement: Supplementary file 5 — Additional file 5: Figure S5. Flowchart for SMA genetic testing and counseling. [file 13023_2024_3523_MOESM5_ESM.tif]
