# Supplementary material for: Unraveling the genetic mysteries of spinal muscular atrophy in Chinese families
Source: Orphanet J Rare Dis. 2025 Jan 15;20:25. doi: 10.1186/s13023-024-03523-0 (PMC11734480; doi:10.1186/s13023-024-03523-0)
Supplement: Supplementary file 6 — Additional file 6. [file 13023_2024_3523_MOESM6_ESM.docx]

Supplementary Table 1 The details of each primer.

| Primer name | Sequence(5' to 3') | Fragment length |
| --- | --- | --- |
| SMN-F | GTTGGGGGATCAAATATCTTCTAGTGTT | 28.2 kb |
| SMN-R | CCCCCACCCCAGTCTTTTACAGATGGT |  |
| Ex1-F | GCGAGGCTCTGTCTCAAAACA | 439 bp |
| Ex1-R | GATCGACTTGATGCTGTCCCGA |  |
| Ex2a-F | CACATAACCTCTAACCAGGTAA | 396 bp |
| Ex2a-R | GGAGGATATCACCTGATTTAACT |  |
| Ex2b-F | GGTGTATGATGCCTTTAAGAGCAGTTT | 555 bp |
| Ex2b-R | CTTCTCCCTGCCTTCCATTCACA |  |
| Ex3-F | GCACCATACGCATTTTATCTC | 690 bp |
| Ex3-R | GAAACTTGGCTTTCATTTTCATTC |  |
| Ex4-F | TTCAATTTCTGGAAGCAGAGA | 383 bp |
| Ex4-R | CAAAAGTTTCATGGGAGAGC |  |
| Ex5-F | GACTTCAGGATTTGGTACATGA | 354 bp |
| Ex5-R | CCCAAGGGATGTTCTACAATGAC |  |
| Ex6-F | CAACATAGCAAGACCTCGTCT | 431 bp |
| Ex6-R | TGCAAGAGTAATTTAAGCCTCAGA |  |
| Ex7-F | GCTCCAGGTCTCAAGTGAT | 680 bp |
| Ex7-R | GTGCAGTATGCCTAGGTTAT |  |
